# Long Term Physiologic and Behavioural Effects of Housing Density and Environmental Resource Provision for Adult Male and Female Sprague Dawley Rats

**DOI:** 10.3390/ani7060044

**Published:** 2017-06-01

**Authors:** Christopher J. Pinelli, Francesco Leri, Patricia V. Turner

**Affiliations:** 1 Department of Pathobiology, University of Guelph, Guelph, ON N1G 2W1, Canada; christopher.joseph.pinelli@emory.edu; 2 Department of Psychology, University of Guelph, Guelph, ON N1G 2W1, Canada; fleri@uoguelph.ca

**Keywords:** environmental enrichment, social housing, fecal corticoid metabolites

## Abstract

**Simple Summary:**

The effect of housing on rodent well-being has received increasing scrutiny in recent years in North America in an attempt to define adequate standards of animal care. Typically, the adequacy of cage size is determined by species and body weight, with little consideration being given to the cage complexity and impact on animal welfare. Further, most studies evaluate different caging methods over relatively short periods of time, which may not be realistic for typical colony settings. Inappropriate housing may lead to boredom, aggression, and the development of abnormal behaviour and function, which in turn may affect animal responses during research protocols. In this study, we evaluated the impact of group housing rats by sex in double wide cages with the addition of polyvinyl chloride (PVC) tubes for shelter, a nylon chew toy, and a piece of food enrichment given three times weekly over the course of five months. These groups were compared to rats that were single or pair-housed by sex in standard cages provided only with a nylon chew toy. We found that group-housed rats were more active and continued to engaged in positive social behaviours, such as grooming, for the duration of the study, with minimal impact on other aspects of animal function.

**Abstract:**

There is considerable interest in refining laboratory rodent environments to promote animal well-being, as well as research reproducibility. Few studies have evaluated the long term impact of enhancing rodent environments with resources and additional cagemates. To that end, male and female Sprague Dawley (SD) rats were housed singly (*n* = 8/sex), in pairs (*n* = 16/sex), or in groups of four (*n* = 16/sex) for five months. Single and paired rats were housed in standard cages with a nylon chew toy, while group-housed rats were kept in double-wide cages with two PVC shelters and a nylon chew toy and were provided with food enrichment three times weekly. Animal behaviour, tests of anxiety (open field, elevated plus maze, and thermal nociception), and aspects of animal physiology (fecal corticoid levels, body weight, weekly food consumption, organ weights, and cerebral stress signaling peptide and receptor mRNA levels) were measured. Significant differences were noted, primarily in behavioural data, with sustained positive social interactions and engagement with environmental resources noted throughout the study. These results suggest that modest enhancements in the environment of both male and female SD rats may be beneficial to their well-being, while introducing minimal variation in other aspects of behavioural or physiologic responses.

## 1. Introduction

The effect of the caging paradigm on rat behaviour has not been well studied, particularly as it relates to the routine and long-term management of rats in animal research facilities. In North America, rats are typically housed according to body weight, with recommendations for post-weaned rats ranging from 185 cm^2^ to 250 cm^2^; cage sizes only five times bigger than those required for mice, despite that fact that rats can be 10–25 times larger [1,2]. It may be assumed that rats do not require a lot of space in research facilities because naturally, they live in small, interconnected underground burrows. Further complicating the issue is that environmental enrichment is poorly defined as a concept when it comes to rats, and can include social elements, as well as those that are nonsocial (e.g., structural modifications, opportunities for exercise, play, and exploration, and food foraging). While the goal of optimizing housing for research animals should be to promote positive affective states to ensure a good life by enhancing social interactions, as well as improving environmental resources [3], it can be difficult to evaluate the effects of environments on rats, since they are so behaviourally plastic and adapt readily to many environments [4]. Realistically, limiting the enclosure size to the minimum required for a good animal condition is necessary to maximize rat housing density in a facility and to minimize caging costs in research facilities. Recent studies have suggested that rats would prefer to live in groups of up to five in larger cages, rather than in pair-housed conditions [5,6]. However, these studies were relatively short in duration and evaluated few physiologic or behavioural parameters, other than the strength of individual rat preference, so it is difficult to fully interpret these findings and suggests that further long term studies are needed to assess the benefit.

Numerous studies have reported the beneficial effects of environmental enrichment on rat learning, affective responses, basal physiology (e.g., corticosterone levels), enhanced immune responsiveness, decreased pain responsiveness, and an enhanced ability to cope with novel and potentially stressful situations (for reviews, see [7,8]). Many of these studies have evaluated extremes in housing conditions, for example, individually housed rats in suspended stainless steel cages versus large enclosures of group housed animals on a substrate provided with numerous food treats, manipulanda, and cage furniture, all changed at regular intervals [9,10,11]. While interesting for evaluating the general effects on behaviour, these environments provide too much variability, unsurmountable sanitation challenges, and unreasonable cage size requirements to be practically implemented within a typical research facility. For environmental enhancements to work within a facility, they should ideally not interfere with the research being conducted, should be easy and inexpensive to maintain, should not be toxic or dangerous to the animal, and should be readily sanitizable. For that reason, readily sanitizable polyvinyl chloride (PVC) tubes and nylon bones were used in this study. 

Suboptimal housing, especially over long periods of time, may trigger reactions of stress, distress, and adverse affective states including anhedonia, anxiety, and aggression in rodents [12]. These states in themselves have all been shown to adversely affect the response to painful stimuli via the induction of hyperalgesia, decrease learning, stimulate aggression, and reduce immune responsiveness following disease challenge. For this reason, chronic stress is an undesirable variable when maintaining rodents in a research facility. The main components of the stress response system are the hypothalamic-pituitary-adrenal gland (HPA) axis and the locus coeruleus/norephinephrine system. Within the HPA axis, potential threats are conveyed to the hypothalamus, which releases corticotrophin-releasing hormone (CRH) via neurons in the paraventricular nucleus. Two types of CRH receptors exist, CRHR1 and CRHR2 (both soluble and insoluble forms) [13], and these are located in varying levels throughout the brain, including the cerebral cortex, amygdala, and diencephalon [14]. Manual restraint, also perceived as acute stress, results in increased CRH mRNA expression [15,16]. CRHR1 mRNA downregulation is also known to occur in response to acute stress in rats [17]. CRH acts on the pituitary, influencing the secretion of adrenocorticotropic hormone (ACTH). ACTH acts rapidly to induce the release of glucocorticoids (corticosterone in rodents) from the adrenal glands. These, in turn, bind to specific receptors within the central nervous system (CNS), turning off the hormonal stress response and restoring a steady state.

Pro-opiomelanocortin (POMC) is a peptide precursor to ACTH and its expression is another indicator of HPA activation. One study found that POMC mRNA expression was decreased in rats undergoing immobilization stress for a duration of three weeks [18]. Conversely, a previous study demonstrated increased POMC mRNA expression in the rat paraventriculate nucleus after one hour of immobilization stress [19]. These findings suggest that long term HPA activation may result in different POMC mRNA levels than short term activation. Orexin, a peptide known to activate the HPA axis, was found to be significantly decreased after acute restraint stress in male rats housed in isolation for 20 days compared to group-housed animals [20]; a finding which is consistent with an observed decrease in the HPA response and suggestive of HPA blunting due to social isolation. Most studies evaluating chronic stress in rats have done so over a relatively short period of time (up to five weeks) [21,22]. In rodents, fecal corticosterone levels are a useful and noninvasive means of monitoring arousal and the basal metabolic state in rats [23]. 

In a previous study, we demonstrated that long term housing of single or pair housed rats in standard cages resulted in blunting of the normal Circadian pattern of corticosterone levels [4]. This occurred despite frequent positive human attention and led us to question whether current management practices for rats are optimal and whether corticosterone blunting is a natural mechanism whereby domesticated rats adapt to a relatively bland cage environment. This has also led us to question whether these rats are experiencing anhedonia or increased anxiety. We believe that these long-term changes in corticosterone levels in rats may have a significant relevance in terms of metabolism, learning, pain response, and basic physiology over time and is therefore an important line of research to pursue.

Our hypothesis is that the long term housing of male and female Sprague Dawley (SD) rats under standard and relatively barren husbandry conditions may result in increased agonistic behaviour and chronic stress. While this may be an adaptive coping mechanism, it may also lead to an altered experimental animal, both behaviorally and physiologically, when they are maintained for use in long term studies. Our objectives were to evaluate the effects of providing an increased cage size, group size, and mildly enhanced cage complexity in preventing these effects over a five-month period. Measures of stress included fecal corticosterone levels, brain CRH and related peptide mRNA levels, and terminal organ and body weights. The impact on affective behaviour was evaluated via behavioural observation, tests of anxiety including open field testing and elevated plus maze [24], and thermal nociception [25]. The five-month time frame allowed for an applicable view of these parameters over the lifespan of a more typical study period.

## 2. Materials and Methods

### 2.1. Study Design

Young adult male (*n* = 80) and female (*n* = 80) Sprague Dawley rats were purchased from Charles River Laboratories (St Constant, QC, Canada) at five weeks of age. Vendor surveillance records indicated that the rats were free of known bacterial, viral, and parasitic pathogens, including the pneumonia virus of mice, sialodacryoadenitis virus, Kilham rat virus, Toolan H1 virus, rat parvovirus, reovirus 3, Theiler murine encephalomyelitis virus, lymphocytic choriomeningitis virus, *Bordetella bronchiseptica*, *Corynebacterium kutscheri*, *Helicobacter hepaticus*, *Helicobacter bilis*, *Clostridium piliforme*, *Salmonella* spp., *Streptobacillus moniliformis*, *Streptococcus pneumoniae*, *Pseudomonas aeruginosa*, *Streptococcus* β *hemolytica*, *Klebsiella pneumoniae*, *Pasteurella* spp., *Mycoplasma pulmonis*, and common ecto- and endoparasites. 

**Figure 1:**
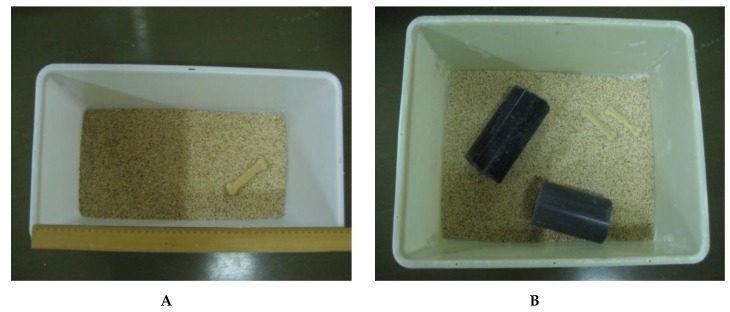
Cage configurations for single or pair-housed rats (**A**) vs. group-housed rats (**B**).

Rats were randomized on arrival and housed singly (*n* = 8/sex), in isosexual pairs (*n* = 8 pairs/sex), or in isosexual groups (*n* = 4/sex/group × 4 groups/sex), in polysulfonate cages (44.5 cm × 24.5 cm (1090 cm^2^) for single or paired rats and 47.5 cm × 38 cm cages (1805 cm^2^) for group-housed rats with wire lids on hardwood chip bedding at 21 ± 2 °C (Figure 1). For the initial two weeks prior to study initiation, rats were acclimated to a 12:12-h reverse-lighting cycle (lights on at 1900). Food (Global 2014 Chow, Harlan, Indianapolis, IN, USA) and water were provided ad libitum throughout the five-month study. Environmental resources consisted of a nylon chew toy for single and pair-housed animals and two PVC tubes, a nylon chew toy, and three times weekly hand feeding of a single piece of toasted oat cereal (Cheerios^®^, General Mills, Mississauga, ON, Canada) per animal for group-housed animals. Individual rats were identified on the tail by a felt marker. The facilities and procedures are in compliance with the Animals for Research Act of Ontario and the Guidelines of the Canadian Council on Animal Care. The University of Guelph Animal Care Committee approved the study protocol (08R047).

### 2.2. Clinical Observations, Body Weight, and Food Consumption

Individual body weights were collected weekly and clinical observations were recorded daily (Table 1). For food consumption, because some animals were paired or group-housed, food consumption was recorded as an average per animal. Distributed food was given per cage at the beginning of the week and the remaining food in the hopper was weighed seven days later to determine the total amount eaten per week. Food consumption data for Week 8 are absent because of a recording error. Discrepancies in food consumption were noted in Weeks 7 and 9, and were attributed to experimental recording error by a new laboratory assistant. Facility fire alarm testing occurred at the end of Week 9 of the study; however, no critical testing was being conducted at this time. For all testing, the order of testing by cage was determined using a random number generator (www.random.org).

### 2.3. Behavioural Observations

Animals were directly observed in their home cages for 30-min periods within the first 2 h of the dark phase at Weeks 3, 5, 7, 11, and 16. Behaviours were scored manually using scan sampling and an ethogram, based on 15 s time intervals (see Table 2). Categories recorded on the ethogram included food or water directed, self-grooming, other behaviour (e.g., stretching, yawning, sniffing), cage-directed (e.g., sniffing cages walls), social behaviour (nonaggressive, e.g., allogrooming), agonistic behaviour (fighting, chasing, submission, vocalization), inactive or resting states (either alone or with another), enrichment-directed (e.g., burrowing, chewing toy, in tunnel), and abnormal behaviour (e.g., bar biting, self-chewing). No a priori assumptions were made about various behaviours when ethogram data were collected, because this process was intended to yield a general understanding of how and whether rat behaviour was altered by the housing paradigm.

### 2.4. Open Field Test

Locomotor activity was monitored in 12 semi-transparent Plexiglas chambers (30 × 40 × 26 cm; University of Guelph, Guelph, ON, USA) lit by individual LED lights (42 diodes) and covered by a black wire mesh to allow video tracking with EthoVision (v11.5, Noldus, Wageningen, The Netherlands). The distance moved was defined as the total horizontal movement (cm). On each test day, animals were introduced into the centre of the chambers and locomotion was recorded for 60 min.

### 2.5. Elevated Plus Maze

The elevated plus-maze was a plus-shaped, black Plexiglas maze with two opposing open arms (50 × 10 cm), two enclosed arms (50 × 10 × 40 cm), and a middle platform (10 × 10 cm). It was elevated 50 cm above the floor and illuminated by a dim light. On each test day, each rat was placed individually in the middle platform, facing towards an open arm, and was allowed to explore the maze for five min. Time spent in the enclosed arms, middle platform, and open arms was recorded by video tracking with EthoVision (v11.5, Noldus, Wageningen, The Netherlands).

### 2.6. Thermal Nociceptive Testing

Nociceptive responses were evaluated prestudy and during Weeks 4, 8, 14, 17, and 20 by using a 50 °C hotplate test (model No. LE7406, Letica Scientific Instruments, Barcelona, Spain). This hotplate temperature is not associated with thermal injury in rats. The evaluation was run as a two-day test, and reaction times were measured 30 min after administering saline (1 mL/kg SC) on day 1 or morphine (1 mg/kg SC; morphine sulfate, Sandoz, Boucherville, QC, Canada) on day 2. Testing occurred during the first 4 h of the dark phase. The endpoint used was licking or shaking a paw or jumping. An arbitrary cutoff time of 80 s was adopted. If no endpoint was achieved, a latency of 80 s was assigned, at which time the rat was removed from the hotplate.

### 2.7. Fecal Corticoid Metabolites

For fecal corticoid metabolite (CORT) determination at Weeks 1, 5, and 10, all feces produced in a light or dark period over 12 h was collected and weighed. Samples were frozen at −20 °C until extracted. Extraction followed a published technique [26]. Briefly, samples were dried for 2 h at 30 °C, weighed, pulverized, and a 0.2 g sample was removed for extraction. To the fecal sample, 0.8 mL water and 5 mL of dichloromethane were added, and the samples were vortexed for 30 s total in 5 s pulses. Following this, the samples were centrifuged for 15 min at 1690× *g*. The bottom dichloromethane fraction was transferred and washed with 1 mL of 0.1M NaOH by vortexing for 10 s, followed by centrifugation for 10 min at 1690× *g*. The dichloromethane fraction was transferred and washed twice with water, centrifuged, and again transferred to a fresh tube. Of the final dichloromethane fraction, 1 mL was transferred and evaporated to dryness under N_2_ for approximately 15 min and was then stored at −20 °C until analysis. Samples were re-suspended in 1 mL of 95% ethanol, vortexed, and diluted to 1:25 with a kit assay buffer. The CORT concentration was determined using the Correlate EIA Kit (Assay Designs, Ann Arbor, MI) according to manufacturer’s instructions. ELISA plates were read at 405 nm (PowerWave XS, BioTek, Winooski, VT, USA). The concentration was determined as % bound using a standard curve ranging from 32 pg/mL to 20,000 pg/mL (kit sensitivity is 27 pg/mL). Values were expressed relative to the total feces collected over a time period and were also evaluated as ng corticosterone/g of feces. The assay kit has a 28.6% cross-reactivity with deoxycorticosterone/desoxycorticosterone, metabolites of corticosterone. Thus, the values measured and reported largely represent corticosterone and these metabolites. While the term “fecal corticoid metabolites” more accurately reflect the assay outcome, the term total corticosterone (CORT) has been used in figures for the sake of brevity. All samples were run in duplicate, and samples from different test periods were randomized to ELISA plates. 

The intra-assay coefficient of variation was 3.3% and the inter-assay coefficient of variation was 5.8%. A linear regression performed on the standard concentration to percent corticoid bound curve demonstrated excellent linearity (R^2^ ≥ 0.99% with an standard deviation (SD) of 0.003%). 

### 2.8. Euthanasia, Relative Organ Weights, and Tissue Collection

At the end of the study, all rats were euthanized by CO_2_ inhalation. Terminal body weights were recorded prior to euthanasia and the relative brain, liver, and paired adrenal gland weights were determined. The hypothalamus and pituitary gland were dissected from the brains for mRNA extraction, placed into RNA*later* (Thermo Fisher Scientific, Mississauga, ON, Canada), and frozen at −80 °C pending analysis. 

### 2.9. Brain mRNA Analyses

For mRNA analyses, the hypothalamus and pituitary samples were separately homogenized and the total mRNA was extracted using an RNeasy Lipid Mini-Kit (Qiagen Sciences, Mississauga, ON, Canada), in accordance with the manufacturer’s instructions, and quantified (ND-1000 spectrophotometer, NanoDrop Technologies, Wilmington, DE, USA). cDNA was synthesized (First Strand cDNA Synthesis Kit for RT-PCR, Roche Applied Science, Laval, QC, Canada) using 1 μg of starting total RNA using the primers listed in Table 3, obtained from Sigma-Aldrich (Sigma-Genosys, Oakville, ON, Canada). The reaction conditions were optimized using traditional PCR and agarose gel electrophoresis. Real-time PCR was performed using the LightCycler 480 System (Roche Applied Science, Laval, QC, Canada) and LightCycler 480 SYBR Green I Master (Roche Applied Science, Laval, QC, Canada) using 20 μL volumes, and the conditions were as follows: initial denaturation at 95 °C for 5 min, 45 cycles of denaturation at 95 °C for 20 s, 20 annealing time using a primer-specific annealing temperature, and extension at 72 °C for 20 s, followed by melting curve analysis. For GAPDH, the annealing temperature was set to 60 °C, and for the CRH receptor subtypes, 63 °C was used. Relative quantitative analysis was done using LightCycler software, comparing the target:reference ratios (both GAPDH and *Β*-actin used as reference genes) and standard curve analysis for the efficiency calculation. Melting curve analysis was utilized to determine the quality of the PCR sample, in addition to running electrophoresis on all real-time samples on gels made from 2% agarose (Ammersham Biosciences, Uppsala, Sweden) in 0.5 × TBE.

### 2.10. Data Analysis 

Because the effect of the environment was the primary factor of interest, and because rats were paired or grouped within environments, statistical evaluations were conducted at the cage level. Data were initially evaluated for normality and homogeneity of variance (sphericity). For all data, planned comparisons were achieved by evaluating the effects of environmental resources in each sex-social housing group [27]. ANOVA was used to assess the effects of environmental resources, sex, social grouping, and time on body weight, food consumption, behaviour, elevated plus maze, locomotor activity, thermal nociception, fecal corticoid metabolites, organ weight ratios, and mRNA expression. Holm-Bonferroni correction was applied to the comparison series to account for multiple comparisons [28]. Post hoc Tukey tests were used when significant interactions were found. Significance was set at a p value less than 0.05. Data were analyzed using SPSS 23.0 software (IBM, Chicago, IL, USA). 

## 3. Results

### 3.1. Body Weight and Food Consumption

**Figure 2:**
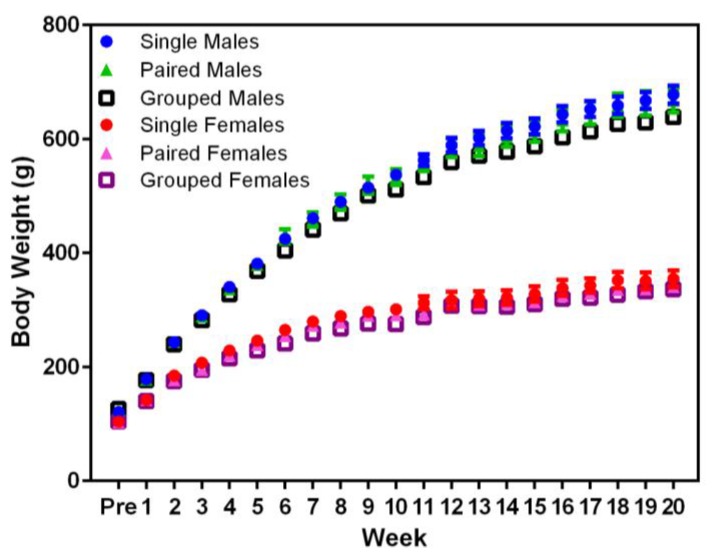
Effect of housing density and environment on mean (±SE) body weight (g) over time in male and female Sprague Dawley (SD) rats.

Planned comparisons indicated that animal body weight did not not differ significantly between housing conditions in males or females (Figure 2). Significant differences were noted in the body weight between sexes, with males weighing more than females (F_1,36_ = 717.71, *p* < 0.001) from Week 1, and both sexes weighed more over time (F_20,720_ = 2555.54, *p* < 0.001).

**Figure 3:**
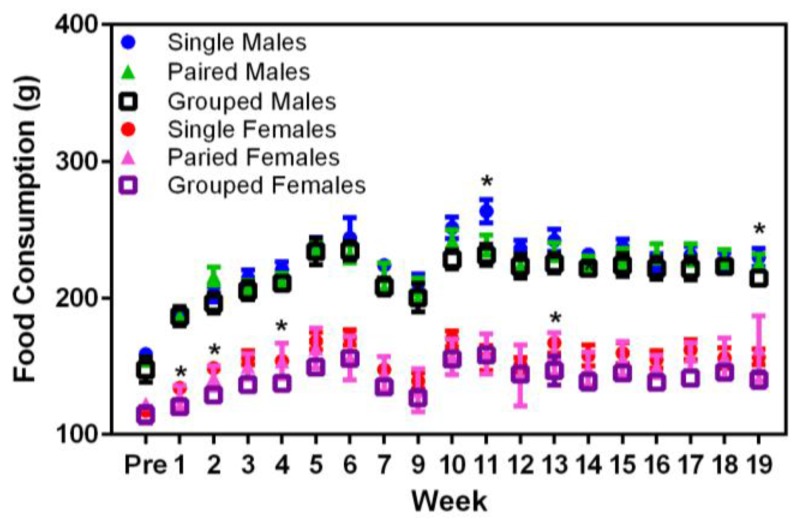
Effect of housing density and environment on mean (±SE) food consumption (g) over time in male and female SD rats.

Significant main effects were seen in the food consumption between sexes (F_1,36_ = 374.68, *p* < 0.001), where males consumed more food than females (Figure 3). In Week 9, singly housed male rats displayed higher food consumption values than either the pair- or group-housed males (F_18,324_ = 18.87, *p* < 0.001). Similarly, in Weeks 2, 4, and 13, singly housed female rats consumed more food than either the pair- or group-housed females (F_18,324_ = 26.70, *p* < 0.001). Given that these differences only occurred at random timepoints in the middle of the study and there were no concurrent changes in the body weights of these rats at the same times, it may be that the differences noted in food consumption were due to reasons other than direct consumption, e.g., food wastage.

### 3.2. Clinical Observations

Rats in the group-housed condition all readily adapted to the food resource and 100% (32 of 32) of rats accepted the oat cereal each time it was offered throughout the study period. All rats survived to the end of the study and no rats in the pair or grouped housing paradigm required separation from cagemates because of aggression. One single-housed female had a focal superficial skin ulceration that persisted variably for the duration of the study. The lesion was responsive to intermittent topical steroid-antibiotic ointment (nystatin-neomycin sulfate-thiostrepton-triamcinolone acetonide, Panalog, Elanco, Guelph, ON, Canada) treatment. Nylon chew bones in group-housed cages of both sexes were consistently well-worn and required periodic replacement throughout the study, compared to less used and worn bones from singly housed rats. Group-housed rats of both sexes used the tubes supplied in various ways, climbing over them, moving or lying between them and, frequently, resting partially or completely within a tube or sometimes with two rats partially in either end of the same tube.

Approximately halfway through the study (at Week 12), 75% (six of eight) of singly housed male rats demonstrated a poorly groomed, unkempt appearance (yellow cast to the pelage and more porphyrin staining on the face and pelage), and 25% (two of eight) had consistently increased periorbital porphyrin-staining (chromodacryorrhea) compared with pair-or group-housed male rats and all female rats in any housing condition. In general, male rats in all groups were less meticulous in appearance, in terms of overall grooming, compared with female rats. Singly-housed rats of both sexes were noted to be easier to handle and restrain with less demonstrated struggling than pair-or group-housed animals. Group-housed animals were more active and resistant to being handled, and were more active and exploratory when placed in new environments (e.g., scale for weekly body weights) than single or paired animals. Twenty-five percent (four of 16) of paired females, 18.75% (three of 16) of paired males, and 10% (two of 20) of group-housed males exhibited barbering, resulting in focal superficial alopecia, with two rats (one single-housed female and one group-housed male) demonstrating self-barbering (based on a forepaw pattern of barbering). Allogrooming was commonly observed in pair- and group-housed rats of both sexes.

### 3.3. Observation of Homecage Behaviour

**Figure 4:**
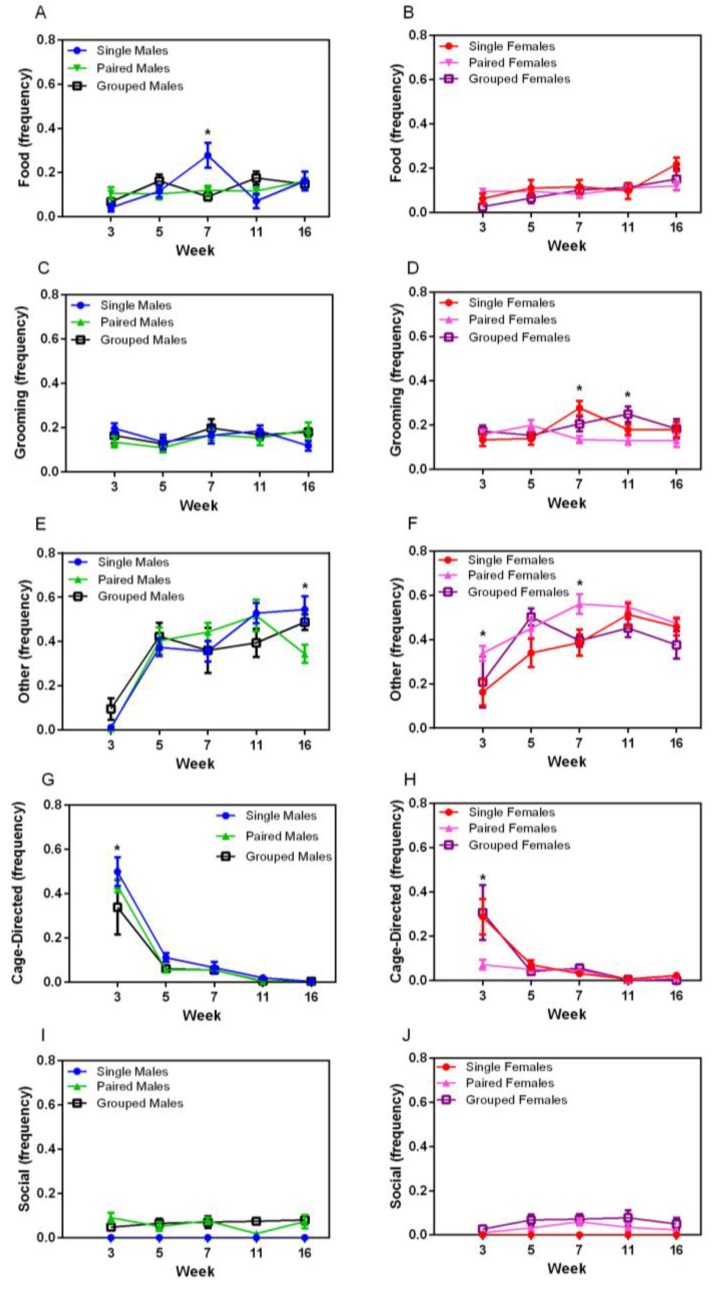
Effect of environment on (**A**,**B**) food and water consumption, (**C**,**D**) grooming, (**G**,**H**) cage, and (**E**,**F**) other behaviors, and (**I**,**J**) social behaviours in male and female rats, respectively, housed singly, in pairs, or in group housing with various cage resources over time. * *p* < 0.008 between groups.

Food- and water-directed behaviour gradually increased over time in both sexes (F_4,144_ = 4.89, *p* = 0.001) (Figure 4A,B). Temporary housing differences were noted at Week 7 in male rats, in which singly housed males exhibited significantly more food and water directed behaviour (F_8,72_ = 3.32, *p* < 0.003) compared with other groups of male rats. Grooming behaviour varied over time, but with no obvious pattern (Figure 4C,D). No sex differences were noted in grooming behaviour, but significant main effects were noted for housing for female rats (F_2,18_ = 3.98, *p* = 0.037), with group-housed animals grooming more than those in pairs (*p* = 0.048). Significant main effects for time and housing were noted in females at Week 7, with single-housed females grooming more than pairs (*p* = 0.002). Other behaviour (stretching, yawning, sniffing) increased over time for all animals (F_8,144_ = 4.04, *p* = 0.004) (Figure 4D,E), with a significant interaction between week and housing groups (F_8,144_ = 2.72, *p* = 0.008). No differences were seen in males except at Week 16, in which paired males demonstrated significantly less of this behaviour than other male groups (F_8,72_ = 38.76, *p* < 0.001), whereas paired females demonstrated significantly more of this behaviour compared with other groups (F_2,18_ = 6.51, *p* = 0.007) at Weeks 3 and 7 (*p* < 0.008). 

Cage-directed behaviours varied significantly over time (F_4,144_ = 84.49, *p* < 0.001 for males and F_4,144_ = 20.54, *p* < 0.001 for females) (Figure 4G,H), with a decrease between weeks 3 and 5 and a subsequent plateau, except for pair-housed females. There was no significant housing main effect. Social behaviour did not vary over time for pair- or group-housed male or female rats, with animals demonstrating allogrooming and other forms of positive interactions throughout the duration of the study (Figure 4I,J). 

**Figure 5:**
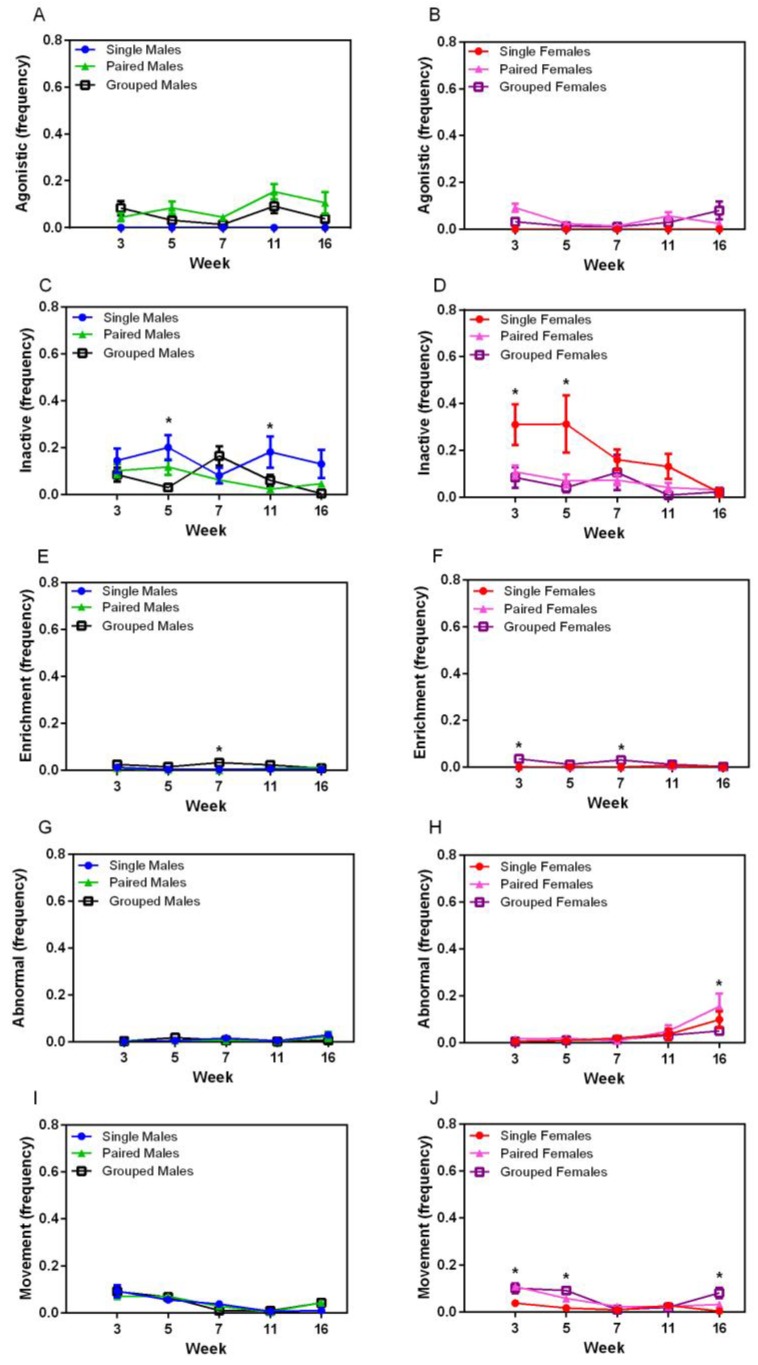
Effect of environment on (**A**,**B**) agonistic behaviour, (**C**,**D**) inactivity, (**E**,**F**) enrichment directed activity, (**E**,**F**) abnormal behaviour, and (**I**,**J**) movement in male and female rats, respectively, housed singly, in pairs, or in group housing with various cage resources over time. * *p* < 0.008 between groups.

A significant interaction was seen for the agonistic behaviour between sexes (F_1,36_ = 8.67, *p* = 0.006), with males demonstrating more agonistic behaviour over the course of the study than females (Figure 5A,B). Agonistic behaviour varied significantly over time, with no distinct pattern, and there was no effect of housing on agonistic behaviour. Inactive behaviour significantly varied over time (F_4,144_ = 4.92, *p* < 0.007) (Figure 5C,D), and a housing effect was seen (F_2,36_ = 9.33, *p* < 0.001) where single-housed animals showed more inactive behaviour than those in pairs (*p* < 0.002) or groups (*p* < 0.003). Significant main effects of housing were seen for inactive behaviour in males at Week 5 (F_2,18_ = 8.259, *p* = 0.001) and Week 7 (F_2,18_ = 6.977, *p* = 0.002), and in females at Week 5 (F_2,18_ = 8.818, *p* = 0.001). Males housed in groups showed less inactivity than pairs (*p* = 0.004) at Week 5 and grouped males were more inactive than pairs at Week 7 (*p* = 0.003). Single-housed females showed more inactivity than pairs (*p* = 0.003) or groups (*p* = 0.001) at Week 5, but no significant differences were noted at Week 7. Significant housing effects were present for enrichment-directed behaviour (F_2,36_ = 15.30, *p* < 0.001), where group-housed animals of both sexes exhibited more of this behaviour than single- (*p* < 0.001) or pair-housed (*p* < 0.001) animals (Figure 5E,F). Significant main effects were seen in males at Week 7 (F_2,18_ = 6.73, *p* = 0.007), with group-housed animals exhibiting this behaviour more than pair-housed animals (*p* = 0.009). Abnormal behaviour occurred very infrequently in this study, but was exhibited more in males than females (F_1,36_ = 9.64, *p* < 0.004) (Figure 5G,H). Significant differences were seen over time in females (F_4,72_ = 9.45, *p* < 0.001) with a sharp increase between weeks 11 and 16, but this was not significantly different between housing groups. Movement and locomotion behaviours decreased significantly over time (F_4,144_ = 48.06, *p* < 0.001) for all animals, and an interaction between sex and housing group was noted (F_2,36_ = 6.36, *p* < 0.001), with a housing main effect in females (F_2,36_ = 11.88, *p* < 0.001), where single-housed females demonstrated physical activity significantly less than pair- (*p* = 0.001) and group-housed (*p* < 0.001) females.

### 3.4. Open Field Testing

**Figure 6:**
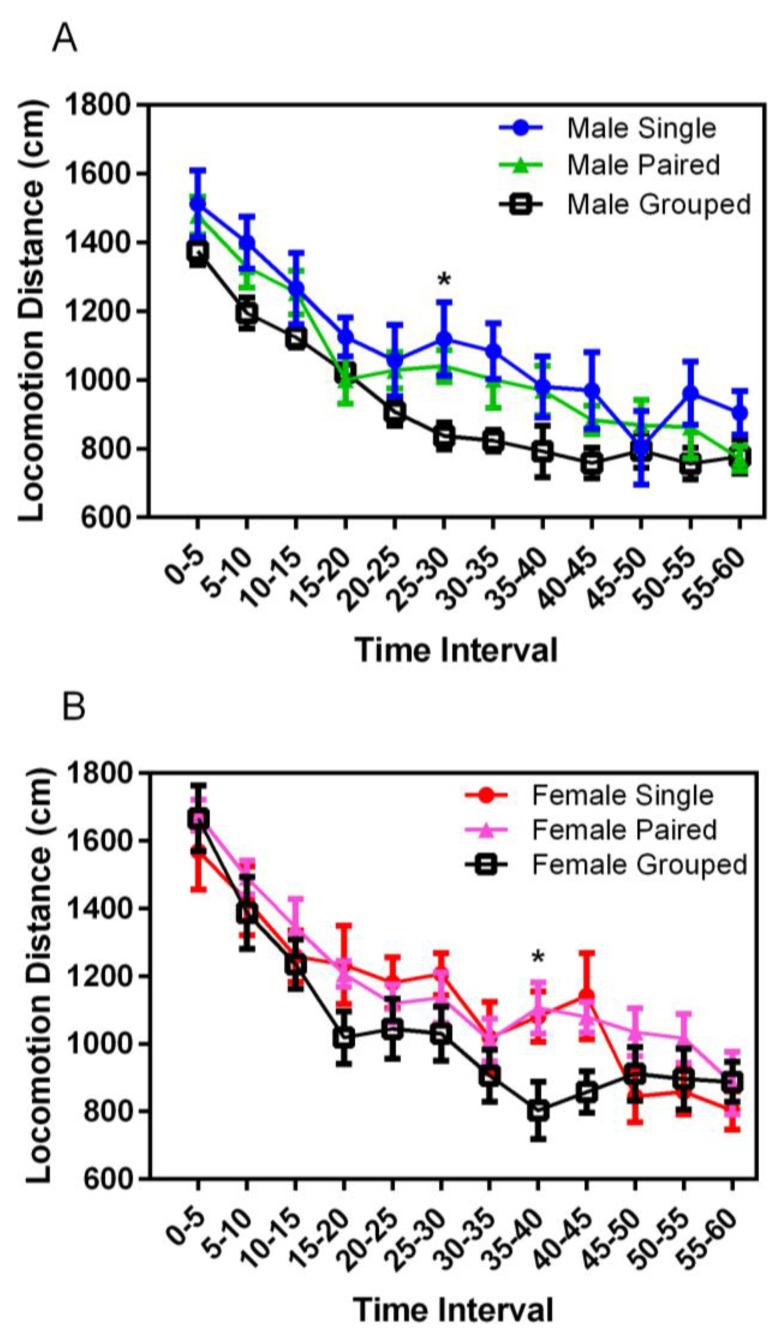
Effect of housing density and environment on mean (±SE) locomotor activity (cm) over a 60 min trial in an open field test chamber in male (**A**) and female (**B**) SD rats.

Locomotor activity in a novel environment decreased significantly over the duration of the chamber experience for both males and females (F_11,398_ = 81.59, *p* = 0.001) (Figure 6). While not consistently significant, group-housed animals of both sexes demonstrated decreased activity after the first 5 min of exploration, compared with both singly- and pair-housed animals; significant for group-housed males at 25–30 s (*p* < 0.001) and for group-housed females at 35–40 s (*p* < 0.001). No significant differences were noted for the total distances traveled based on housing or sex.

### 3.5. Elevated Plus Maze

**Figure 7:**
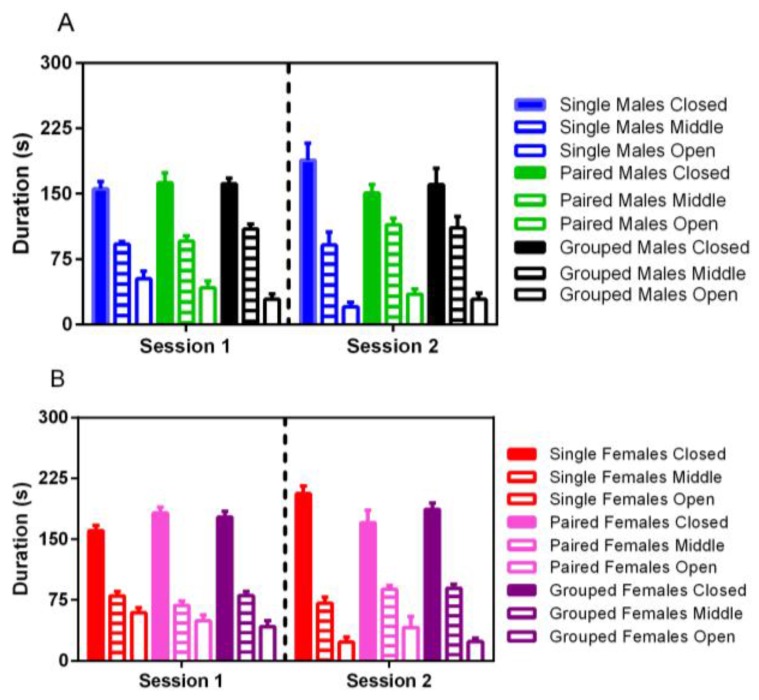
Effect of housing density and environment on mean (±SE) duration of time (s) spent in the open, closed, and middle part of an elevated plus maze in male (**A**) and female (**B**) SD rats.

No significant differences were noted for elevated plus activity based on housing, although male rats spent significantly more time in the middle portion of the maze in both trials than females (F_1,35_ = 22.86, *p* < 0.001) (Figure 7). For both males and females, less time was spent in the open arm of the maze in the second trial (F_1,35_ = 14.38, *p* < 0.001). 

### 3.6. Thermal Nociceptive Testing

**Figure 8:**
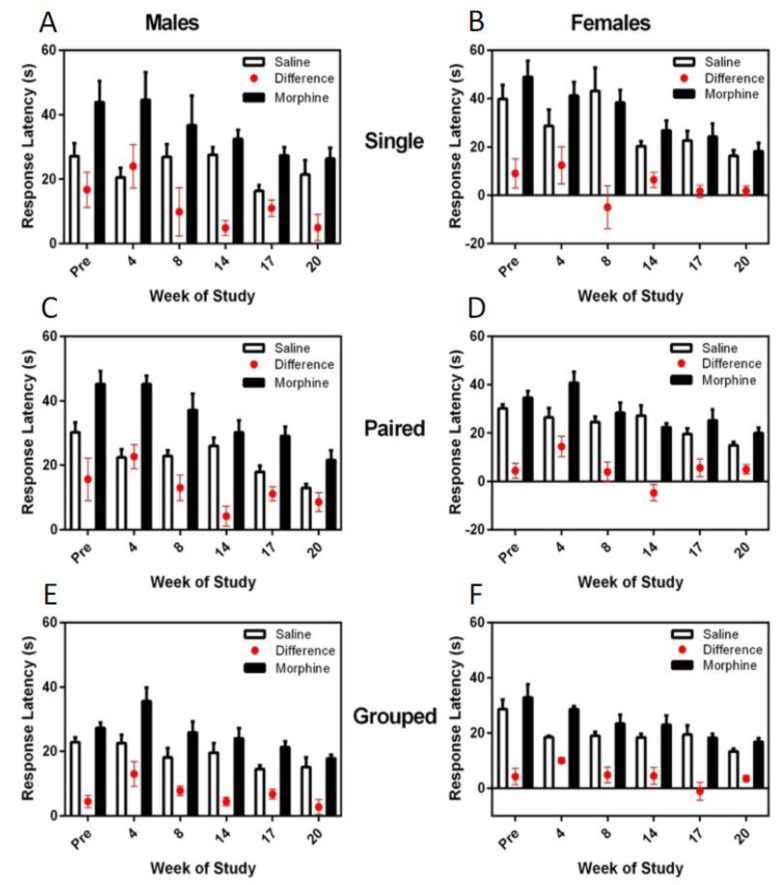
Effect of housing density and environment on mean (±SE) response latency (s) during thermal nociceptive testing in male and female SD rats housed singly (**A,B**), in pairs (**C,D**) or in groups (**E,F**), respectively. Studies were conducted over two days with morphine or saline administered S.C. approximately 30 min before testing. Latency differences were calculated by subtracting the saline latency from the morphine latency.

The difference in hotplate latency between saline and morphine treatment was consistently and significantly greater in males (F_1,36_ = 8.48, *p* = 0.006), and for both males and females, the latency difference between the morphine and saline response times decreased over the trials (F_5,180_ = 7.16, *p* < 0.001) (Figure 8). Housing environment had no effect on the hotplate latency responses; however, hotplate responses were significantly more consistent and faster for group-housed animals than for single- or pair-housed rats.

### 3.7. Fecal Corticoid Metabolites

**Figure 9:**
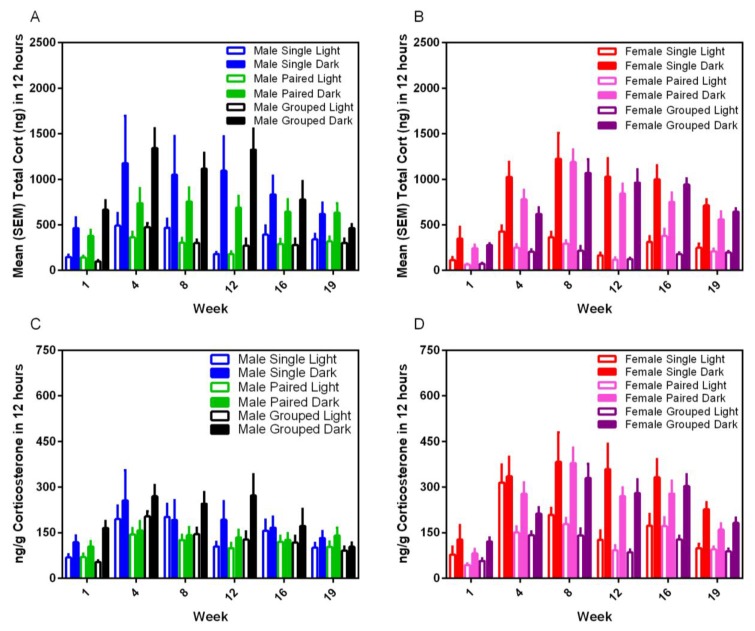
Effect of housing density and environment on mean (±SE) fecal corticoid levels (total (**A**,**B**) and ng/g feces (**C**,**D**)) in male and female SD rats, respectively. For each sampling date, all feces present in each cage was collected for the entire duration of each 12 h light or dark period prior to extraction.

Housing environment had no effect on fecal corticoid metabolite levels for either sex or strain and in either phase of the photoperiod.; however, dark cycle measurements were always at least three–five-fold higher than for the light cycle (F_1,35_ = 123.15, *p* < 0.001) (Figure 9). Corticosterone levels varied significantly over time (F_5,175_ = 19.08, *p* < 0.001) with peaks in the total CORT between Weeks 4 and 12 for males, and 4 and 16 for females, and a gradual blunting in levels in all groups over subsequent weeks.

### 3.8. Organ:Body Weight Ratios

A significant sex difference was noted for brain:body weight ratios (F_1,21_ = 127.96, *p* < 0.001), with females (0.0057 ± 0.0006) having a higher ratio than males (0.0032 ± 0.0003). No other sex or housing differences were noted for liver or paired adrenal gland relative weights. 

### 3.9. Brain mRNA Expression

There were no differences in hypothalamic *CRH* mRNA expression for either sex; however, relative pituitary *CRHR1* mRNA levels were significantly decreased in paired male rats compared to single-housed males (*p* = 0.012) (Table 4). In addition, the relative mRNA expression for *OR2* was significantly increased in pair-housed females compared to single-housed females (*p* < 0.023).

## 4. Discussion

Clinical and behavioural observations proved to be of the greatest value for assessing the long-term impact of altering the cage size, animal number, and addition of modest environmental resources in male and female SD rats. These findings are in keeping with the results of a similar study we conducted in male and female mice, assessing the long-term impact of additions of environmental resources on animal welfare [28]. In the current study, single-housed male rats were poorly groomed, with more perinasal porphyrin staining by three months, suggesting that the effects of social isolation were amplified by sexual maturation. Single-housed animals were also more docile when handled than pair- or group-housed animals, which may be an indication of learned helplessness; a consequence of other stressors, such as inescapable foot shock [29,30]. More barbering was seen in paired females and males over time compared to group-housed females and males, which is indicative of social stress [29] and tends to corroborate previous studies suggesting that rats prefer to be housed in small groups [5]. Similarly, the behavioural data suggested that group-housed rats have higher levels of activity and demonstrated consistent engagement with conspecifics and environmental resources over the duration of the five-month study. Singly-housed male and female rats demonstrated more inactivity, which may be consistent with a higher level of boredom compared to other housing paradigms. While, in general, lower levels of locomotion and exploration in the open field test are consistent with increased levels of anxiety in rats [24], the fact that male and female group-housed rats demonstrated a similarly high level of initial exploration as the single- and pair-housed groups suggests that the more rapid reduction in exploratory behaviour may have been due to a more rapid habituation and perceived loss of novelty in these groups. This phenomenon has been reported for the open field test by others evaluating environmental enrichment in rats [31]. Similar to Brenes et al. [31], there was no effect of the minor enhancement of the cage environment on elevated plus maze outcomes.

While no differences were noted between housing groups for either sex for thermal nociceptive latency, group-housed animals showed decreased morphine and saline latency times compared to single- and paired-, or just single-housed animals, respectively. Other studies have demonstrated that more anxious rodents demonstrate mild hyperalgesia and thus longer hotplate latencies in response to saline [32] and this is an area that may require further work.

The fecal corticoid metabolite concentrations measured in the current study are consistent with those of previous studies, with significantly higher levels of excretion during the dark phase [4]. No sex differences were noted in the levels in this study. While female rats excrete higher levels of corticosterone than males, plasma protein binding and excretion patterns may vary between animals, such that higher levels are not necessarily reflected in fecal corticoid assays [33]. Similar to our previous findings [4], blunting in nocturnal levels of fecal corticoids were noted in both male and female rats after approximately three to four months of the study. It is unknown whether this is a consequence of decreased activity in rats held in inadequate environments with time and is an area for further exploration.

*CRHR1* mRNA data showed that male rats had the least mRNA expression when housed in pairs, and the highest relative expression when singly-housed. Previous studies have demonstrated that stress results in the decreased mRNA expression of *CRH* receptors [17,34]. This may suggest that the pair housing of male rats in barren environments without visual hides or shelters, may be more stressful than single housing. Further, levels of orexin mRNA expression were noted to be lowest in singly-housed female rats, compared with pair- or group-housed female rats. This suggests that this housing state may be chronically stressful for female rats. Given the numerous gender differences seen in response to variations between other experimental variables, it would not be surprising if there were differential housing preferences between male and female SD rats, and this is an area for further study.

The most significant limitation of this study was that the housing and environmental resource changes for the group-housed rats likely represent an incremental change from standard conditions, and thus, may not be expected to induce major shifts in physiology or homeostasis. Other than behavioural assessments, which can be challenging to do consistently, particularly over long periods of time, the tools that are available to study the impact of modest environmental changes on affective states, such as organ:body weight ratios or thermal nociceptive sensitivity, are somewhat crude, limiting our availability to gather evidence about cause and effect relationships on animal comfort or other positive states.

## 5. Conclusions

In conclusion, we have demonstrated that simple environmental improvements combined with small group social housing provide subtle long term enhancements to male and female SD rats, for example, by reducing stereotypic behaviours, increasing activity levels, and providing opportunities for allogrooming. At the same time, these improvements had a minimal impact on animal physiology, such as body weight and food consumption patterns, and the outcomes of standard anxiety and nociception assays. To ensure positive welfare states and a good life for research animals, it is important that advancements are made in rodent husbandry in step with scientific knowledge. The environmental changes that were introduced in this study were inexpensive and modest in nature, and are well within the financial reach of many facilities housing rats. Despite the beneficial effects noted, it is apparent that rats quickly habituate to all environments and vivarium routines unless they are regularly changed, leading to the blunting of circadian corticosterone levels over time. Our results also suggest that housing density preference in SD rats may vary by sex.

## Figures and Tables

**Table 1:** Experimentation schedule throughout study. Not depicted are body weight and food consumption, which were recorded weekly. Abbreviations: Fec Cort-Fecal Corticosterone Collection, Beh Obs-Behavioural Observation, HP-Hotplate Testing, E+-Elevated Plus Maze Testing, Loc-Open Field Testing.

Wk 1	Wk 2	Wk 3	Wk 4	Wk 5	Wk 6	Wk 7	Wk 8	Wk 9	Wk 10	Wk 11	Wk 12	Wk 13	Wk 14	Wk 15	Wk 16	Wk 17	Wk 18	Wk 19	Wk 20
Fec Cort			Fec Cort				Fec Cort				Fec Cort				Fec Cort			Fec Cort	
		Beh Obs		Beh Obs		Beh Obs				Beh Obs					Beh Obs				
			HP		E+		HP					E+	HP			HP			
								Loc											

**Table 2:** Ethogram used for scoring the affective behaviour in rats.

Category	Description
Food or water directed	Eating, drinking
Grooming	Self-grooming
Other	Stretching, yawning, sniffing
Cage-directed	Sniffing cages walls
Social	Nonaggressive, allogrooming
Agonistic	Fighting, chasing, submission, vocalization
Inactive (or resting)	Not moving, sleeping
Enrichment-directed	Burrowing, chewing toy, in tunnel
Abnormal	Bar biting, self-chewing
Movement	Locomotion with no apparent other incentive

**Table 3:** mRNA primer sequences used for RT-PCR ^a^.

Gene	Forward Primer	Reverse Primer
*CRHR1*	5′-TTCACCTCCCTTCAGGATCA-3′	5′-TGCAGGCCAGAAACATTGC-3′
*CRHR2insoluble*	5′-TTTGGATGACAAGCAGAGGAAGT-3′	5′-GCACTAGGAAAAGCAGGAAAGC-3′
*CRHR2soluble*	5′-CCCATTTTGGATGACAAGGAGTA-3′	5’-GGATGAAGGTGGTGATGAGGTT-3′
*CRH*	5′-CAGAACAACAGTGCGGGCTCA-3′	5′-AAGGCAGACAGGGCGACAGAG-3′
*POMC*	5′-GAGATTCTGCTACAGTCGCTC-3′	5′-TTGATGATGGCGTTCTTGAA-3′
*PPO*	5′-CATCCTCACTCTGGGAAAG-3′	5′-AGGGATATGGCTCTAGCTC-3′
*OR1*	5′-GCGATTATCTCTATCCGAAGC-3′	5′-CAGGGACAGGTTGACAATG-3′
*OR2*	5-′TGTTCAAGAGCACAGCCAAACG-3′	5′-GCCAATACCATAAGACACAGGGG-3′
*GAPDH*	5′-AAGGGCTCATGACCACAGT-3′	5′-GGATGCAGGGATGATGTTCT-3′
*Β-actin*	5′-TTGCTGACAGGATGCAGAA-3′	5′-ACCAATCCACACAGAGTACTT-3′

^a^ CRHR: corticotropin releasing hormone receptor, CRH: corticotropin releasing hormone, POMC: pro-opiomelanocortin, PPO: prepro-orexin, OR: orexin receptor, GAPDH: glyceraldehyde 3-phosphate dehydrogenase. CRH receptor subtype and GAPDH primers were developed based on sequences from [17]. CRH and β-actin primers were based on sequences derived from [22]. POMC primers were derived from [18]. Pre-pro-orexin, OR1, OR2, primers were derived from C. Kotz (personal communication).

**Table 4:** Effect of housing density and cage environment on brain mRNA expression for various hypothalamic and pituitary gland factors expressed relative to housekeeping genes, *GAPDH*, and *β-actin* in male and female SD rats.

mRNA	Males				Females			
	Single (Mean ± SD)	Paired (Mean ± SD)	Grouped (Mean ± SD)	ANOVA (F, *p*-Value)	Single (Mean ± SD)	Paired (Mean ± SD)	Grouped (Mean ± SD)	ANOVA (F, *p*-Value)
*CRH GAPDH*	0.2923 (0.1722)	0.4472 (0.1258)	0.5372 (0.0059)	F = 2.543, *p* = 0.128	0.4507 (0.3929)	0.4958 (0.2773)	0.7980 (0.1820)	F = 0.806, *p* = 0.476
*CRH β-actin*	0.2386 (0.1697)	0.3506 (0.1077)	0.3860 (0.0060)	F = 1.122, *p* = 0.363	1.5425 (2.6600)	0.4104 (0.2154)	0.6917 (0.1160)	F = 0.423, *p* = 0.667
*CRHR1 GAPDH*	0.0803a (0.0248)	0.0276a (0.0132)	0.0535 (0.0029)	^a^ F = 6.774, *p* = 0.014	0.0458 (0.0194)	0.0557 (0.0142)	0.0261 (0.0042)	F = 2.104, *p* = 0.178
*CRHR2ins GAPDH*	0.0271 (0.0313)	0.0016 (0.0004)	0.0068 (0.0005)	F = 6.774, *p* = 0.014	0.0150 (0.0137)	0.0191 (0.0100)	0.0025 (0.0009)	F = 1.364, *p* = 0.304
*CRHR2sol GAPDH*	0.0089 (0.0101)	0.0010 (0.0003)	0.0111 (0.0113)	F = 1.008, *p* = 0.399	0.0028 (0.0033)	0.0019 (0.0006)	0.0005 (0.0001)	F = 0.627, *p* = 0.556
*POMC GAPDH*	16.1315 (11.2131)	8.7327 (0.9728)	8.0022 (0.6740)	F = 1.035, *p* = 0.390	10.4343 (9.5909)	8.0516 (6.1742)	5.1556 (0.2989)	F = 0.351, *p* = 0.713
*POMC β-actin*	6.839 (5.4718)	3.1873 (0.2556)	3.3894 (0.5108)	F = 0.936, *p* = 0.424	4.3849 (3.8119)	3.5791 (3.4275)	2.1285 (0.5048)	F = 0.324, *p* = 0.731
*PPO GAPDH*	0.2432 (0.5318)	0.4823 (0.1597)	0.6233 (0.1665)	F = 0.711, *p* = 0.515	0.4359 (0.4116)	0.5073 (0.3328)	0.6083 (0.0185)	F = 0.178, *p* = 0.840
*PPO β-actin*	0.0920 (0.2028)	0.2016 (0.0815)	0.2696 (0.1227)	F = 0.998, *p* = 0.403	0.1806 (0.1615)	0.2109 (0.1424)	0.2475 (0.0004)	F = 0.169, *p* = 0.847
*OR1 GAPDH*	1.9038 (0.4468)	2.2582 (0.3352)	2.5676 (0.0922)	F = 2.510, *p* = 0.131	2.0782 (0.8748)	2.6174 (1.1895)	2.1880 (0.1400)	F = 0.398, *p* = 0.683
*OR1 β-actin*	0.7810 (0.2302)	0.8246 (0.1607)	1.1141 (0.1177)	F = 2.046, *p* = 0.180	0.9529 (0.5778)	1.1557 (0.7081)	0.8870 (0.0359)	F = 0.191, *p* = 0.829
*OR2 GAPDH*	3.1750 (0.8673)	3.0825 (0.3883)	3.5322 (0.2223)	F = 0.237, *p* = 0.793	2.7934b (0.3253)	3.4439b (0.2906)	3.2330 (0.2428)	^b^ F = 5.719, *p* = 0.025
*OR2 β-actin*	1.2943 (0.3841)	1.1837 (0.0741)	1.4911 (0.2380)	F = 0.517, *p* = 0.611	1.2409 (0.03791)	1.4643 (0.3410)	1.3102 (0.0716)	F = 0.505, *p* = 0.620

Post-hoc *p*-Value: ^a^ *CRHR1 GAPDH*: a- *p* = 0.012; ^b^ *OR2 GAPDH*: b- *p* = 0.023.
